# BPOZ-2 is a negative regulator of the NLPR3 inflammasome contributing to SARS-CoV-2-induced hyperinflammation

**DOI:** 10.3389/fcimb.2023.1134511

**Published:** 2023-03-02

**Authors:** Jingfei Li, Haotian Lin, Tinghui Fan, Linfei Huang, Xinyong Zhang, Yanhong Tai, Yi Fang, Qihong Li, Ruzhou Zhao, Penghao Wang, Li Zhou, Luming Wan, Yuhua Wu, Hui Zhong, Congwen Wei, Xiaopan Yang

**Affiliations:** ^1^ Department of Genetic Engineering, Beijing Institute of Biotechnology, Beijing, China; ^2^ Department of Medical Oncology, Beijing Tuberculosis and Thoracic Tumor Research Institute/Beijing Chest Hospital, Capital Medical University, Beijing, China; ^3^ Department of Pathology, Fifth Medical Center of Chinese PLA General Hospital, Beijing, China; ^4^ Department of Endocrinology, Fifth Medical Center of Chinese PLA General Hospital, Beijing, China; ^5^ Department of Stomatology, Fifth Medical Center of Chinese PLA General Hospital, Beijing, China

**Keywords:** BPOZ-2, NLPR3, inflammasome, SARS-CoV-2, hyperinflammation

## Abstract

**Introduction:**

Inflammation play important roles in the initiation and progression of acute lung injury (ALI), acute respiratory distress syndrome (ARDS), septic shock, clotting dysfunction, or even death associated with SARS-CoV-2 infection. However, the pathogenic mechanisms underlying SARS-CoV-2-induced hyperinflammation are still largely unknown.

**Methods:**

The animal model of septic shock and ALI was established after LPS intraperitoneal injection or intratracheal instillation. Bone marrow-derived macrophages (BMDMs) from WT and BPOZ-2 KO mouse strains were harvested from the femurs and tibias of mice. Immunohistology staining, ELISA assay, coimmunoprecipitation, and immunoblot analysis were used to detect the histopathological changes of lung tissues and the expression of inflammatory factors and protein interaction.

**Results and conclusions:**

We show a distinct mechanism by which the SARS-CoV-2 N (SARS-2-N) protein targets Bood POZ-containing gene type 2 (BPOZ-2), a scaffold protein for the E3 ubiquitin ligase Cullin 3 that we identified as a negative regulator of inflammatory responses, to promote NLRP3 inflammasome activation. We first demonstrated that BPOZ-2 knockout (BPOZ-2 KO) mice were more susceptible to lipopolysaccharide (LPS)-induced septic shock and ALI and showed increased serum IL-1β levels. In addition, BMDMs isolated from BPOZ-2 KO mice showed increased IL-1β production in response to NLRP3 stimuli. Mechanistically, BPOZ-2 interacted with NLRP3 and mediated its degradation by recruiting Cullin 3. In particular, the expression of *BPOZ-2* was significantly reduced in lung tissues from mice infected with SARS-CoV-2 and in cells overexpressing SARS-2-N. Importantly, proinflammatory responses triggered by the SARS-2-N were significantly blocked by BPOZ-2 reintroduction. Thus, we concluded that BPOZ-2 is a negative regulator of the NLPR3 inflammasome that likely contributes to SARS-CoV-2-induced hyperinflammation.

## Introduction

Severe acute respiratory syndrome coronavirus 2 (SARS-CoV-2) belongs to the Coronaviridae family and β-coronavirus genus and has caused a worldwide pandemic in over 220 countries (Wang et al., 2021). SARS-CoV-2 infection causes a range of mild to severe symptoms associated with acute lung injury (ALI), acute respiratory distress syndrome (ARDS), metabolic acidosis, septic shock, clotting dysfunction, or even death. This complex clinical syndrome was designated coronavirus disease 2019 (COVID-19) (Chen et al., 2020). Cytokine storms characterized by elevated levels of multiple cytokines may contribute to the high mortality rate from COVID-19 ARDS (Que et al., 2022). Moreover, NLRP3 inflammasome activation correlates with the severity of disease in COVID-19 patients (Rodrigues et al., 2021) and plays a central role in this excessive inflammatory response (Ratajczak and Kucia, 2020; Shah, 2020; Pan et al., 2021). SARS-CoV-2 has a positive-sense single-stranded RNA genome of approximately 30 kilobases long and encodes 4 structural proteins, 16 nonstructural proteins (nsp1 to 16) and at least 8 accessory proteins (Brant et al., 2021). A previous study suggested that SARS-CoV-2 E protein has dual effects on initial immunosuppression and subsequent NLRP3 inflammasome activation (Yalcinkaya et al., 2021). In addition, the SARS-2-N protein activates NLRP3 inflammasomes by facilitating their assembly (Pan et al., 2021). However, the pathogenic mechanisms underlying SARS-CoV-2-induced hyperinflammation are still largely unknown.

The NLRP3 inflammasome is a multiprotein complex consisting of an NLRP3 sensor protein, an apoptosis-associated speck-like protein containing a caspase recruitment domain (ASC) adaptor protein, and a caspase-1 effector protein. The NLRP3 sensor protein contains three domains: the pyrin domain (PYD), nucleotide-binding domain, and leucine-rich repeat domain. In response to RNA virus infection, the NLRP3 PYD interacts with the PYD of the ASC adaptor protein, promotes ASC oligomer formation, and provides a platform for caspase-1 activation. Active caspase-1 then leads to the secretion of IL-1β by catalyzing pro-interleukin (IL)-1β into mature IL-1β (Swanson et al., 2019). Although inflammasome activation is critical for pathogen clearance and adaptive immune response induction, persistent and excessive IL-1β production can lead to a “cytokine storm” in acute inflammatory diseases by stimulating systemic inflammation responses mainly through activating nuclear factor-κB (NF-κB) or c-Jun N-terminal kinase pathways (Pan et al., 2021).

In this study, we show a distinct mechanism by which the SARS-2-N protein targets Bood POZ-containing gene type 2 (BPOZ-2) to promote NLPR3 activation. BPOZ-2 is ubiquitously expressed in human organs and plays a role in mediating tumor growth suppression by tumor suppressor phosphatase and tensin homolog deleted on chromosome 10 (PTEN) (Dai et al., 2000; Unoki and Nakamura, 2001). Here, we provided evidence showing that BPOZ-2 is a negative regulator of the NLPR3 inflammasome that contributes to SARS-CoV-2-induced hyperinflammation.

## Materials and methods

### Reagents

Dulbecco’s modified Eagle medium (DMEM, high glucose, 11965092), fetal bovine serum (FBS, 10099141), Lipofectamine™ 2000 (11668019), Pierce™ BCA Protein Assay Kit (23225), and Thermo Scientific RevertAid Mix (M16325) were purchased from ThermoFisher Scientific (Waltham, MA, USA). Protease inhibitor cOmplete™ Cocktail (4693116001) was purchased from Roche (Basel, Switzerland). PerfectStart™ Green qPCR SuperMix (AQ601) was purchased from TransGen Biotech (Beijing, China). NucleoZOL (740404) was purchased from MACHEREY-NAGEL (MN, Düren, Deutschland). Mouse IL-1β ELISA kit (1210122), human IL-1β ELISA kit (1110122), mouse IL-6 ELISA kit (1210602), and mouse TNF-α ELISA kit (1217202) were purchased from DAKEWE (Shenzhen, China). LPS (L2880) was purchased from Sigma (Missouri, USA). ATP (tlrl-atpl) and MSU (tlrl-msu) were purchased from *In vivo*Gen.

### Antibodies

An anti-Flag M2 affinity gel (A2220) and anti-Flag-HRP antibody (A8592) were purchased from Sigma (Missouri, USA). An anti-Myc antibody (AE010), an anti-V5 antibody (AE017) and an anti-β-tubulin antibody (AC030) were purchased from Abclonal (Wuhan, China). An anti β-Actin antibody (66009-1-Ig) was purchased from Proteintech (Wuhan, China). An anti-HA-HRP antibody (14031S) and a normal rabbit IgG antibody (2729s) were purchased from Cell Signaling Technology (Massachusetts, USA). A goat anti rabbit-HRP antibody (ZB-2301) and a goat anti mouse-HRP antibody (ZB-2305) were purchased from ZSGB-BIO (Beijing, China). Protein A/G PLUS-Agarose (sc-2003) was purchased from Santa Cruz Biotechnology (Texas, USA).

### Plasmids and cell culture

Mammalian expression vectors encoding human BPOZ-2, NLRP3, ASC, pro-caspase-1, pro-IL-1β, Cullin 3, and SARS-2-N were constructed by inserting the corresponding PCR-amplified fragments into pcDNA3.1 (Invitrogen, Massachusetts, USA). The A549, THP-1 and HEK293T cell lines were obtained from the American Type Culture Collection (ATCC, Rockville, MD, USA). All cell lines were tested for mycoplasma contamination and were incubated in DMEM at 37°C in a humidified atmosphere with 5% CO_2_. All plasmids and siRNAs were transfected by Lipofectamine™ 2000 following the manufacturer’s protocol. The siRNAs were obtained from GenePharma Co., Ltd. (Shanghai, China). The sequence of *BPOZ-2* siRNA was 5’-UCAACAGCUGCCCUGACAUTT-3’. The sequence of Cullin 3 siRNA was 5’-GCUGCUAUAGUGCGGAUAATT-3’.

### LPS-induced septic shock

The main features of septic shock in mice models are massive production of cytokines, such as IL-1β, TNF-α, and IL-6, leading to inflammatory tissue injuries and finally multiorgan failure. WT, BPOZ-2 HE or BPOZ-2 KO mice were generated by Southern Model Biotechnology (Shanghai, China). Six- to eight-week-old WT, HE, and KO male mice were divided into two groups as follows: intraperitoneal injection of 50 mg/kg LPS for 3.25 h, followed by 50 μL of 100 mM ATP for 15 min. Serum levels of IL-1β, IL-6, and TNF-α were measured immediately by ELISA kits. For mortality studies, septic shock was induced by intraperitoneal injection with 50 mg/kg LPS. The mortality of mice within 36 h was observed, and a survival curve was generated.

### LPS-induced acute lung injury (ALI)

Mice (6–8 weeks of age) were given intratracheal instillation of LPS (5 mg per kg body weight (mg/kg)). The main features of ALI in mice models are rapid onset with accumulation of neutrophils in the alveolar or the interstitial space, thickening of the alveolar wall, increases in total bronchoalveolar protein concentration, and increases in the concentrations of proinflammatory cytokines in lung tissue or BAL fluid. In this model, the BAL fluid was collected and analyzed immediately for cell counts by flow cytometry, protein quantification by BCA Protein Assy Kit, and cytokine analysis by ELISA kits at 6, 24, 48 or 72 h after LPS instillation. Lung tissue was isolated and fixed immediately, embedded in paraffin, and analyzed by staining with hematoxylin and eosin (HE) or immunoblot analysis. For mortality study, mice were treated intratracheally with a high dose (25 mg/kg) of LPS and were observed for up to 7 days.

### Infection with SARS-CoV-2

The mice were infected with the mouse-adapted SARS-CoV-2 strain MASCp6, and the cells were infected by the SARS-CoV-2 strain (BetaCoV/Beijing/IME-BJ01/2020). All the animals and cells infection were conducted in a Biosafety Level 3 laboratory (BSL-3) as previously described (Wan et al., 2022) and were approved by the Animal Experiment Committee of Laboratory Animal Center, Beijing Institute of Microbiology and Epidemiology (approval number: IACUC-DWZX2020-002). Lung tissues were isolated and subjected to *BPOZ-2* RNA analysis or fixed for immunohistochemistry analysis immediately.

### Bone marrow–derived macrophages (BMDMs) isolation

BMDMs from WT and BPOZ-2 KO mouse strains were harvested from the femurs and tibias of mice as described by Rebecca E. Tweedell (Tweedell et al., 2020) with modifications. In brief, cells were cultured in RPMI 1640 medium containing 10% FBS and 20 ng/mL M-CSF. After 7 days of culture, adherent cells were collected and plated in a 12-well plate at a density of 1 × 10^6^ cells per well. LPS-primed BMDMs were stimulated with ATP (5 mM) for 30 min and MSU (200 μg/mL) for 4 h.

### Coimmunoprecipitation and immunoblot analysis

Cells were collected and lysed immediately in NP40 lysis buffer with a protease inhibitor cocktail. Soluble proteins were subjected to immunoprecipitation with an anti-Flag M2 affinity gel or a normal rabbit IgG antibody plus protein A/G-agarose. A sample of the total lysates (5%, v/v) was included as a control. All the proteins used for immunoblot were added with SDS-PAGE loading buffer (with DTT), boiled for 10 min, centrifuged for 2 min at 12,000 g at 4°C, and the supernatants were collected for immunoblot analysis immediately or kept in -80°C. Immunoblot analysis was performed with the appropriate antibody. The antigen-antibody complexes were visualized by a chemiluminescence system.

### Quantitative reverse transcription PCR (RT-qPCR)

Total mRNA was extracted immediately using NucleoZOL as described in “RNA isolation User manual”. In brief, 500 μL NucleoZOL was added per 5 × 10^6^ cells or 50 mg tissue for lysing or homogenizing, and then 200 μL RNase-free water was added to the lysate, shaking, incubating, and centrifuged for 15 min at 12,000 g at room temperature. The supernatant was mixed with isopropanol in equal volume and centrifuged for 10 min at 12,000 g to precipitate RNA. 75% Ethanol was used to wash RNA and RNase-free water was added to dissolve the RNA pellet. The concentration of RNA was measured by nanodrop and its integrity was detected by agarose gel electrophoresis. RNA was used to synthesize cDNA immediately using Thermo Scientific RevertAid Mix, and cDNA was kept in -80°C or mixed with 2 × PerfectStartTM Green qPCR SuperMix (TransGen Biotech) and primers according to the manufacturer’s instructions. PCR was performed for 40 cycles and ABI QuantStudio3 was used to collect SYBR Green I signal. Abs Quant/2nd Derivative Max was used to calculate the Cp value. The relative levels of individual mRNAs were calculated after normalization to the *ACTB* mRNA level. DNase I-treated total RNA was used as a negative control to rule out possible contamination of the RNA sample with genomic DNA. The primer sequences are listed in **Table S1**.

### Statistical analysis

GraphPad Prism 8.0 was used for data plotting and statistical calculations in this study. Differences between two independent samples were evaluated using two-tailed Student’s t tests. Survival rates were analyzed by the log-rank (Mantel−Cox) test. A P value ≤ 0.05 was considered statistically significant. ns (not significant), *P* > 0.05; *, *P* < 0.05; **, *P* < 0.01; ***, *P* < 0.001.

To ensure the main conclusion was not affected by the biases originated from the individual study design, no data were excluded, cell-based studies were performed independently at least three times with comparable results, and all animal experiments were conducted using n=4 or 6 mice.

## Results

### BPOZ-2-deficient mice are more susceptible to LPS-induced septic shock

**Figure 1:**
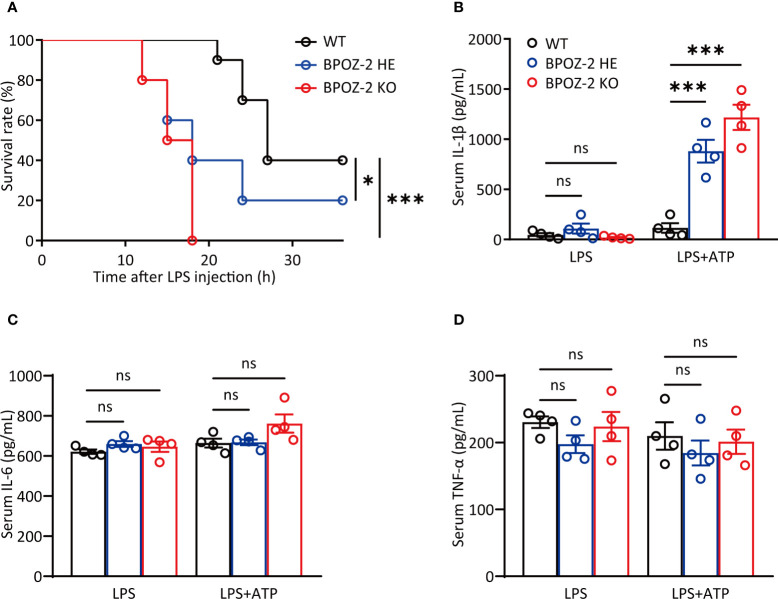
BPOZ-2-deficient mice are more susceptible to LPS-induced septic shock. **(A)** Survival rate of wild-type (WT), BPOZ-2 heterozygous (BPOZ-2 HE) or BPOZ-2 knockout (BPOZ-2 KO) mice (n = 10 per group) after intraperitoneal injection with LPS (50 mg/kg). **(B–D)** The IL-1β **(B)**, IL-6 **(C)**, and TNF-α **(D)** levels of WT, HE or KO mice (n = 4) that were first injected with LPS (50 mg/kg) and then 50 μL 100 mM ATP at 3.25 h after LPS injection. Serum samples were harvested 15 min after the second injection and analyzed by ELISA. Data in **(A)** were analyzed by the log-rank (Mantel−Cox) test. Data in **(B–D)** were analyzed by two-tailed Student’s t tests. ns, not significant, *P<0.05, ***P<0.001.

BPOZ-2 is an adaptor protein for the E3 ubiquitin ligase scaffold protein Cullin 3. BPOZ-2 deficiency decreases sustained activation of hepatic stellate cells and decreases liver fibrosis in acute carbon tetrachloride (CCl4)-induced liver injury, indicating a potential role of BPOZ-2 in inflammation. To assess the involvement of BPOZ-2 in inflammatory responses, we first generated BPOZ-2-deficient (BPOZ-2 KO) and BPOZ-2 heterozygous (BPOZ-2 HE) C57BL/6 mice in which a PGK-Neo cassette replaced a segment from exon 2 to 12 of the BPOZ gene (**Supplementary Figure 1A**). *BPOZ-2* transcripts in multiple tissues were not detected in mutant mice by RT-qPCR (**Supplementary Figure 1B**). We next compared the survival of BPOZ-2 KO and HE mice with WT mice in sepsis mouse models induced with LPS intraperitoneal injection. As was shown, all of the BPOZ-2 KO mice died, whereas none of the WT mice died at 18 h after LPS injection (**Figure 1A**). Importantly, 60% of BPOZ-2 HE mice died, and BPOZ-2 KO mice died far more quickly than their WT littermates and BPOZ-2 HE mice. Therefore, the expression levels of BPOZ-2 are critically important in protecting against LPS-induced sepsis death (**Figure 1A**). We then measured the serum concentration of IL-1β at 9 h after LPS+ATP injection. Notably, more mature IL-1β was produced in BPOZ-2 KO mice than that in their WT littermates and BPOZ-2 HE mice (**Figure 1B**). In contrast, the secretion of IL-6 or TNF-α, which depends on Toll-like receptor (TLR) signaling only, was nearly identical among BPOZ-2 KO, BPOZ-2 HE, and WT mice (**Figures 1C, D**). Together, these data show that BPOZ-2 deficiency results in a greater susceptibility to sepsis, which may be partly due to the increase in serum IL-1β levels.

### BPOZ-2-deficient mice display exacerbated LPS-induced acute lung injury

**Figure 2:**
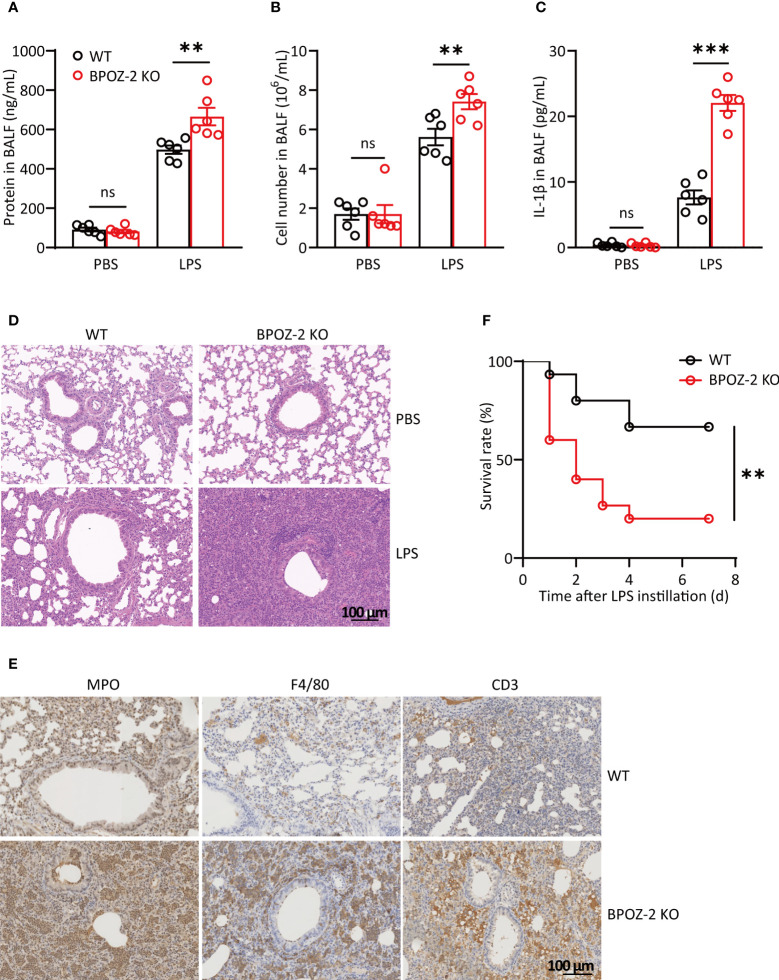
BPOZ-2-deficient mice display exacerbated LPS-induced acute lung injury. **(A–D)** BAL fluid protein concentration **(A)**, total cell counts **(B)**, and IL-1β **(C)** of WT and BPOZ-2 KO mice that were treated with intratracheal 5 mg/kg LPS (n = 6). **(D, E)** Representative images of HE staining **(D)** and immune cells staining **(E)** of the lungs in WT and BPOZ-2 KO mice after 5 mg/kg LPS or PBS instillation. MPO, CD3, and F4/80 antibody was used for neutrophils, lymphocytes, and macrophages staining. Scale bar, 100 μm. **(F)** Survival rate of WT and BPOZ-2 KO mice (n = 15 per group) after intratracheal injection with LPS (25 mg/kg). All images are representative of 3 biological replicates. Data in **(A–C)** were analyzed by two-tailed Student’s t tests. Data in **(F)** were analyzed by the log-rank (Mantel−Cox) test. ns, not significant, **P < 0.01, ***P < 0.001.

Since inflammation also plays important roles in the initiation and progression of acute lung injury (ALI), we next investigated the potential roles of BPOZ-2 in regulating LPS-induced ALI. As reported, the cell content and IL-6 levels in the BAL fluid began to increase at 6 h after LPS instillation (**Figures S2A, B**), whereas the protein content and IL-1β levels were significantly higher at 48 and 72 h after LPS instillation (**Figures S2C, D**). Therefore, the levels of lung inflammatory markers were compared between WT and BPOZ-2 KO mice at 72 h after LPS instillation. The results showed that in the BAL fluid, the total cell and protein contents in BPOZ-2 KO mice were higher than those in WT mice (**Figures 2A, B**). Moreover, BPOZ-2 deficiency also significantly increased serum IL-1β levels in mice (**Figure 2C**). HE staining showed significant tissue damage including neutrophil infiltration, alveolar wall thickening, hemorrhage, and alveolar disruption in the lungs of BPOZ-2 KO mice (**Figure 2D**). BPOZ-2 KO LPS group also had a significantly higher neutrophils, macrophage, and lymphocytes cells infiltration into the lung tissue than that observed in the WT LPS group (**Figure 2E**). Accordingly, BPOZ-2-deficient mice were highly susceptible to death by LPS-induced ALI (**Figure 2F**). These results indicated that BPOZ-2 deficiency remarkably aggravated ALI and pulmonary inflammation during ALI upon LPS challenge.

### BPOZ-2-deficient BMDMs promote IL-1β production induced by NLRP3 stimuli

**Figure 3:**
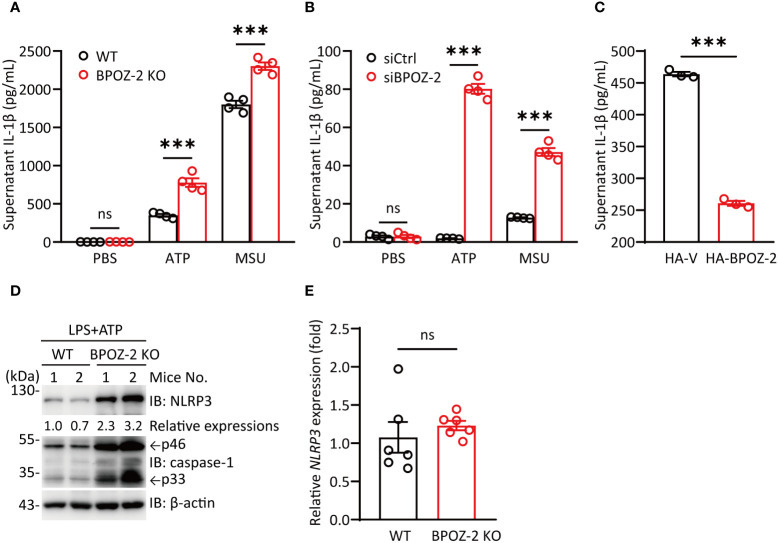
BPOZ-2-deficient BMDMs promote IL-1β production induced by NLRP3 stimuli. **(A)** BMDMs from WT and BPOZ-2 KO mice that were first treated with LPS for 4 h and then treated with 5 mM ATP for 30 min or 200 μg/mL MSU for 6 h. **(B)** THP-1 cells transfected with control siRNA (siCtrl) or *BPOZ-2* siRNA (siBPOZ-2) were treated with LPS and ATP or LPS and MSU as above. Cell supernatants were analyzed by ELISA for IL-1β secretion. **(C)** HEK293T cells were cotransfected with plasmids encoding NLRP3, ASC, pro-caspase-1, pro-IL-1β, and BPOZ-2. After 48 h, cell supernatants were analyzed by ELISA for IL-1β secretion. **(D, E)** *NLRP3*, pro-caspase-1, caspase-1 p33 levels **(D)** and transcript expressions **(E)** in inflammatory signaling pathway in WT and BPOZ-2 KO BMDMs treated with LPS and ATP. All quantified data are presented as the mean ± SEM of n = 3 independent experiments. Data were analyzed by two-tailed Student’s t tests. ns, not significant, ***P < 0.001.

Since BPOZ-2 deficiency significantly increased serum IL-1β levels upon LPS challenge, we next assessed the effects of NLRP3 activators on LPS-primed BMDMs isolated from BPOZ-2 KO mice. Consistent with the *in vivo* data, BPOZ-2 deficiency led to enhanced mature IL-1β secretion in response to NLRP3 activators, including ATP and MSU (**Figure 3A**). In addition, BPOZ-2 expression was knocked down in THP-1 cells using specific siRNA duplexes (**Figure S3A**). Congruent results showed that treatment with *BPOZ-2* siRNA, but not control siRNA, increased mature IL-1β secretion in response to ATP and MSU (**Figure 3B**). Moreover, reduced IL-1β production was observed in THP-1 cells overexpressing BOPZ-2 (**Figure 3C**). By comparing the expression levels of NLRP3 in BMDMs from WT and BPOZ-2 KO mice after LPS and ATP treatment, we found that NLRP3 protein expression was significantly higher in BPOZ-2 KO mice (**Figure 3D**), whereas the transcriptional levels of *NLRP3* were unchanged (**Figure 3E**). Consistently with that, the pro-caspase-1 and its cleaved activated p33 forms were significantly higher in BPOZ-2 KO mice (**Figure 3D**). Therefore, BPOZ-2 may regulate IL-1β production by affecting NLRP3 expression in a posttranscriptional manner.

### BPOZ-2 interacts with NLRP3 and regulates its stability

**Figure 4:**
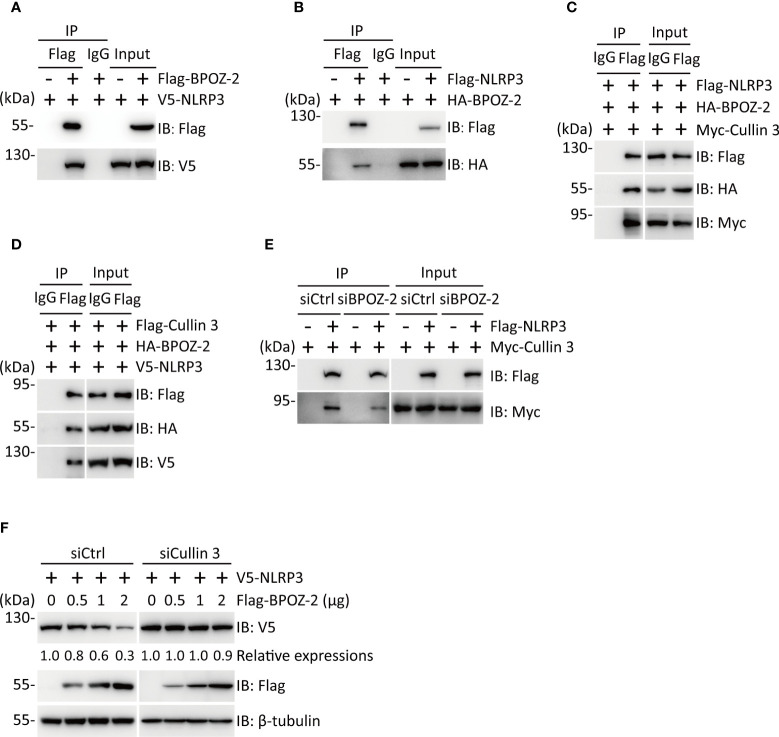
BPOZ-2 interacts with NLRP3 and regulates its stability. **(A, B)** Interaction of BPOZ-2 and NLRP3. V5-NLRP3 was coexpressed with or without Flag-BPOZ-2 in HEK293T cells **(A)**, or HA-BPOZ-2 was coexpressed with or without Flag-NLRP3 in HEK293T cells **(B)**. Immunoprecipitation was performed using the anti-Flag antibody. BPOZ-2 and NLRP3 were detected in the immunoprecipitates and cell lysates by immunoblotting. **(C, D)** Coimmunoprecipitation of BPOZ-2 with NLRP3 and Cullin3. Plasmids encoding Flag-NLRP3, HA-BPOZ-2, and Myc-Cullin 3 or plasmids encoding V5-NLRP3, HA-BPOZ-2, and Flag-Cullin 3 were coexpressed in HEK293T cells and immunoprecipitated from cell lysates using the anti-Flag antibody. BPOZ-2, NLRP3, and Cullin 3 were detected in the immunoprecipitates and cell lysates by immunoblotting. **(E)** HEK293T cells were transfected with *BPOZ-2* siRNA (siBPOZ-2) or siCtrl followed by cotransfecting with plasmids encoding Flag-NLRP3 and Myc-Cullin 3. Immunoprecipitation from cell lysates was performed using the anti-Flag antibody. NLRP3 and Cullin 3 were detected in the immunoprecipitates and cell lysates by immunoblotting. **(F)** HEK293T cells were transfected with Cullin 3 siRNA (siCullin 3) or siCtrl followed by cotransfecting with plasmids encoding V5-NLRP3 and increasing Flag-BPOZ-2. NLRP3 and BPOZ-2 were detected by immunoblotting with the indicated antibodies. β-tubulin was used as equal loading control.

To explore the possible interaction of BPOZ-2 and NLRP3, we performed coimmunoprecipitation experiments in HEK293T cells expressing Flag-BPOZ-2 and V5-NLPR3. Interestingly, BPOZ-2 coimmunoprecipitated with NLRP3 (**Figure 4A**). The binding between BPOZ-2 and NLRP3 was also confirmed in HEK293T cells transfected with Flag-NLRP3 and HA-BPOZ-2 (**Figure 4B**). Since BPOZ-2 is an adaptor protein for the E3 ubiquitin ligase Cullin 3, we wondered that BPOZ-2 might function as an adaptor to recruit Cullin 3 to NLRP3. Coimmunoprecipitation experiments in HEK293T cells transfected with Myc-Cullin 3, HA-BPOZ-2, and Flag-NLRP3 showed that both Cullin 3 and BPOZ-2 were present in the immunoprecipitates of anti-Flag antibody, indicating that NLRP3, Cullin 3 and BPOZ-2 existed in the same complex (**Figure 4C**). Similar results were obtained by transfecting Flag-Cullin 3 with HA-BPOZ-2 and V5-NLRP3 (**Figure 4D**). To ensure that BPOZ-2 was an adaptor for Cullin 3 and NLRP3, we performed similar experiments in *BPOZ-2* siRNA transfected HEK293T cells. As shown in **Figure 4E**, the binding ability of Cullin 3 with NLRP3 was significantly reduced in siBPOZ-2 cells as compared to cells transfected with control siRNA oligos. Therefore, BPOZ-2 was indispensable for the formation of the Cullin 3-BPOZ-2-NLRP3 complex.

Next, we sought to determine the effect of BPOZ-2 on NLRP3 expression. HEK293T cells were transfected with plasmids encoding Flag-NLRP3 together with increasing amounts of plasmid encoding BPOZ-2. The results showed that BPOZ-2 expression led to reduced NLRP3 expression in dose-dependent manner. As expected, BPOZ-2-induced NLRP3 downregulation was nearly abrogated in siCullin 3 cells (**Figures 4F**, **S3B**). Together, our results show that BPOZ-2 serves as a scaffold protein for Cullin 3 regulating NLRP3 protein expression.

### SARS-CoV-2 infection reduces the expression of BPOZ-2

**Figure 5:**
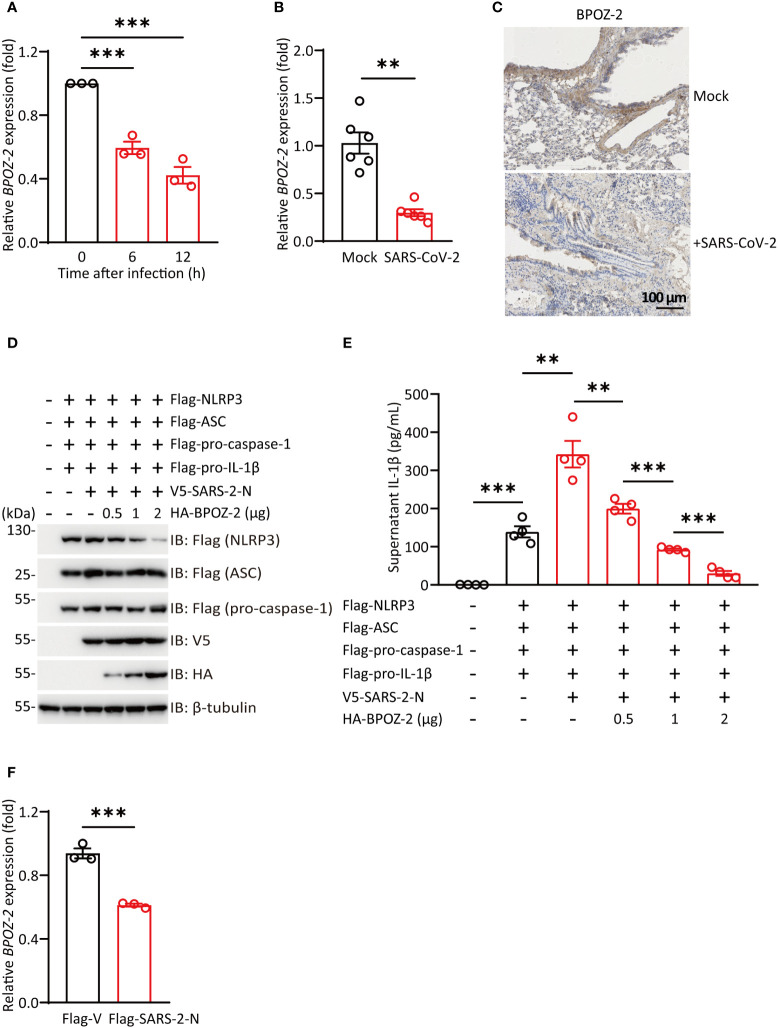
SARS-CoV-2 infection reduces BPOZ-2 expression. **(A)** The relative expression of *BPOZ-2* in Huh-7 cells infected with SARS-CoV-2 for the indicated times. Data are presented as the mean ± SEM of n = 3 independent experiments. **(B)** *BPOZ-2* mRNA expression in lung tissues from mice infected with 1.6 × 10^4^ plaque-forming units of MASCp6 and killed at day 2 after infection (n = 6). **(C)** Representative immunohistochemistry images of BPOZ-2 in the above mouse lungs inoculated with MASCp6. Scale bars, 100 μm. **(D)** HEK293T cells were cotransfected with Flag-NLRP3, Flag-ASC, Flag-pro-caspase-1, Flag-pro-IL-1β, V5-SARS-2-N, and different amounts of BPOZ-2 plasmids or their control plasmid (Flag-V) and cell lysates were immunoblotted using the indicated antibodies. **(E)** IL-1β secretion in reconstituted HEK293T cells transfected with plasmids encoding SARS-2-N protein and different amounts of BPOZ-2 plasmids. **(F)** *BPOZ-2* mRNA expression in cells transfected with Flag-Vector (Flag-V) or Flag-SARS-2-N by RT−qPCR. All images are representative of 3 biological replicates. All quantified data are presented as the mean ± SEM of n = 3 independent experiments except **(A)**. Data were analyzed by two-tailed Student’s t tests. **P < 0.01, ***P < 0.001.

SARS-CoV-2 infection is associated with an excessive inflammatory response and acute respiratory distress syndrome (ARDS). Since BPOZ-2 plays an important role in inflammasome activation in the context of ALI, the question of whether BPOZ-2 is a target of SARS-CoV-2 drew our attention. To examine this possibility, Huh-7 cells were infected with SARS-CoV-2, and *BPOZ-2* expression was examined during the infection. Cultured hepatocytes exposed to SARS-CoV-2 exhibited a robust time-dependent decrease in *BPOZ-2* expression (**Figure 5A**). To further evaluate this finding, we constructed a mouse model infected with the mouse-adapted SARS-CoV-2 strain MASCp6 at passage 621 and compared *BPOZ-2* expression in the lung tissues from MASCp6- or mock-infected mice. On day 2 after infection, reduced *BPOZ-2* expression in the lung tissues was observed in the MASCp6-infected mice (**Figure 5B**). Using immunohistochemistry, we found sharply decreased BPOZ-2-positive staining in multiple lung tissue regions from infected mice (**Figure 5C**), indicating that the expression of BPOZ-2 was reduced by SARS-CoV-2 infection.

### BPOZ-2 downregulation contributes to SARS-CoV-2 hyperinflammation

NLRP3 inflammasome occurred in different types of cells including macrophages, monocytes, and lung epithelial. Reduced BPOZ-2 expression in the lung tissues by SARS-CoV-2 infection implicated that BPOZ-2 might contribute to COVID-19 hyperinflammation. To this end, we reconstituted inflammasomes successfully by transfecting all the components of NLRP3 inflammasome including Flag-pro-IL-1β, Flag-pro-caspase-1, Flag-ASC, and Flag-NLRP3(**Figures 5D, E**). Notably, SARS-2-N transfection sharply stimulated IL-1β production in the reconstituted system (**Figure 5E**). In particular, *BPOZ-2* expression was specifically reduced by SARS-2-N overexpression (**Figure 5F**). Strikingly, reintroduction BPOZ-2 expression to the reconstituted system significantly blocked IL-1β production induced by SARS-2-N in a dose-dependent manner (**Figure 5E**). Therefore, SARS-CoV-2 targets BPOZ-2 to induced inflammasome activation by SARS-2-N.

## Discussion

SARS-CoV-2 infection can cause acute lung injury and cytokine storms as a result of innate immune hyperactivity, which plays a central role in driving ALI in patients with severe COVID-19 (Lee et al., 2020; Lowery et al., 2021). In this study, we demonstrated that hyperinflammation triggered by SARS-CoV-2 infection was partly mediated through BPOZ-2 downregulation by SARS-2-N protein. Several lines of findings support this argument. First, BPOZ-2-deficient mice are more susceptible to LPS-induced septic shock and ALI; BPOZ-2-deficient BMDMs increase mature IL-1β production by NLRP3 inflammasome activation. Second, BPOZ-2-Cullin 3 forms a complex with NLRP3. Cullin 3 is indispensable for BPOZ-2-induced NLRP3 degradation. The expression level of NLRP3 is greatly enhanced in BPOZ-2-deficient BMDMs. Third, *BPOZ-2* expression is greatly reduced upon SARS-CoV-2 infection, especially by SARS-2 N protein. Last, the upregulated inflammasome activation induced by the SARS-2-N protein was greatly reduced by BPOZ-2 reintroduction. As a result of these findings, BPOZ-2 may serve as a negative regulator of the NLRP3 inflammasome, which is hijacked during SARS-CoV-2 infection, highlighting the biological significance of the Cullin 3-BPOZ-2-NLRP3 complex in the delicate control of inflammasome activation.

Proinflammatory cytokines in the IL-1 family are regulators of IL-6 production, and high levels of IL-1β have been associated with cytokine storms and hemophagocytic lymphohistiocytosis (Schulert and Grom, 2015; Zhang et al., 2022). A subset of COVID-19 patients treated with anakinra, a recombinant form of endogenous IL-1Ra that blocks IL-1 activity, experienced a significant reduction in C-reactive protein levels, superior improvement in respiratory functions, and a higher cumulative survival rate compared to patients not treated with anakinra, indicating that the NLRP3 inflammasome is a potential target for COVID-19 therapy (Iglesias-Julian et al., 2020; Kyriazopoulou et al., 2021). In support of this clinical observation, the SARS-2-N protein aggravates lung injury and accelerates cell death in sepsis and acute lung inflammation mouse models by facilitating NLRP3 inflammasome assembly and promoting IL-1β production (Pan et al., 2021). Our results showed that BPOZ-2 deficiency significantly increased serum IL-1β levels and accelerated sepsis-induced cell death. BPOZ-2 reintroduction, however, suppresses the elevated IL-1β production exerted by the SARS-2-N. Therefore, BPOZ-2 plays a protective role in SARS-CoV-2 infection by reversing the cytokine storm. We provide evidence showing that the SARS-2-N protein can directly downregulate *BPOZ-2* transcriptional expression, the mechanisms underlying this phenomenon warrants further investigation. Of particular interest, SARS-CoV-2 infection induces inflammasome-derived PANoptosis, a unique inflammatory cell death pathway including pyroptosis, apoptosis (Kuriakose et al., 2016; Karki et al., 2022), and/or necroptosis regulated by multifaceted PANoptosome complexes (Lee et al., 2021). Given that PANoptosis in SARS-CoV-2 infection may closely correlated with lung injury, multiple organ failure, and unfavorable prognosis in patients with severe COVID-19 (Karki et al., 2021; Lee et al., 2021), it is thus interesting to evaluate the role of BPOZ in PANotosis upon SARS-CoV-2 infection in the future.

In conclusion, our study demonstrates that BPOZ-2 is a negative regulator of NLRP3 inflammasome activation by targeting NLRP3 degradation. Strikingly, the expression of *BPOZ-2* is greatly reduced by SARS-2-N and BPOZ-2 reintroduction significantly blocked NLRP3 inflammasome activation induced by SARS-2-N. Therefore, our findings not only provide novel insight into the roles of BPOZ as a key negative regulator of NLRP3 inflammasomes and clues to the mechanisms underlying the development of SARS-CoV-2-induced hyperinflammation, but also provide a potential therapeutic strategy for patients with SARS-CoV-2 acute respiratory distress syndrome or acute lung injury.

## Data availability statement

The original contributions presented in the study are included in the article/**Supplementary Material**. Further inquiries can be directed to the corresponding author.

## Ethics statement

The animal study was reviewed and approved by Institutional Animal Care and Use Committee and performed at the AMMS Animal Center (Beijing, China).

## Author contributions

XY and HZ designed and drafted the research. JL, HL, TF, LH, XZ, YT, YF, QL, RZ, PW, and LZ participated in the experiments. JL, HL, TF, and LH collected and analyzed the data. LW, YW, HZ, CW, and XY reviewed and revised the manuscript. All authors contributed to the article and approved the submitted version.
