# Supplementary figures and images for: BPOZ-2 is a negative regulator of the NLPR3 inflammasome contributing to SARS-CoV-2-induced hyperinflammation
Source: Front Cell Infect Microbiol. 2023 Mar 2;13:1134511. doi: 10.3389/fcimb.2023.1134511 (PMC10019892; doi:10.3389/fcimb.2023.1134511)

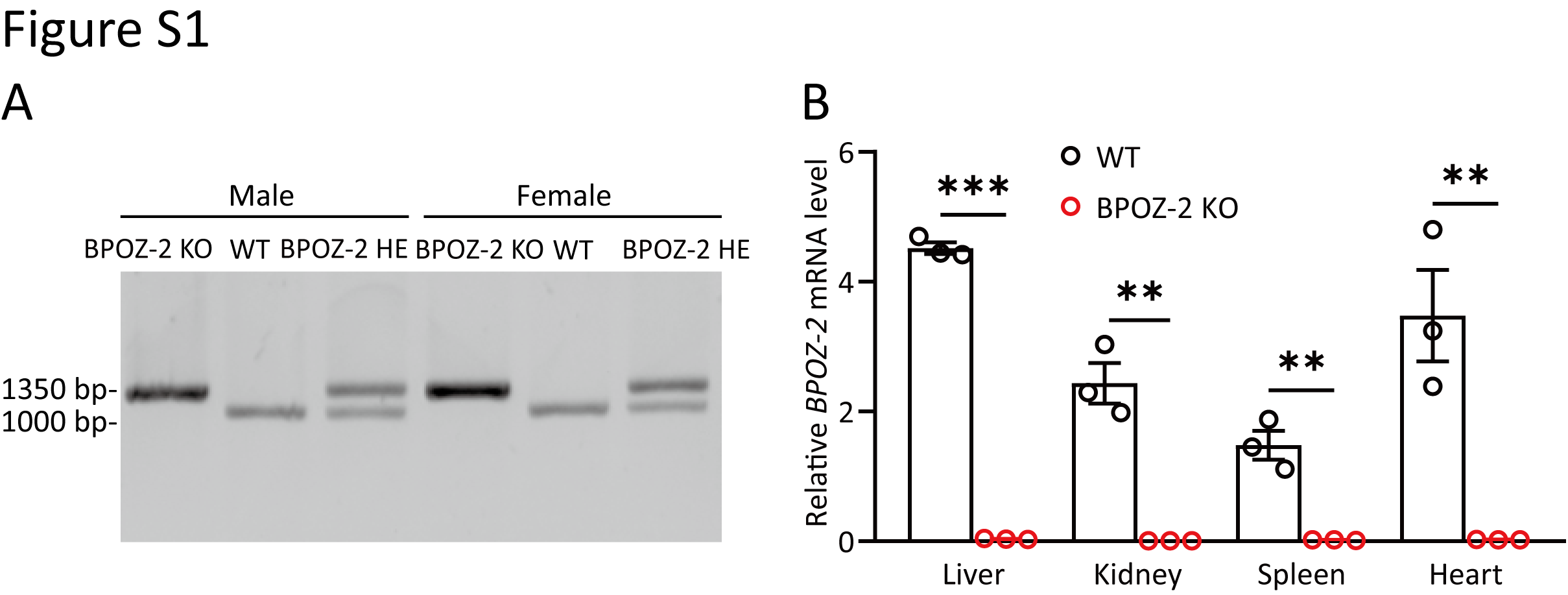

Supplement: Supplementary file 1 [file Image_1.tif]

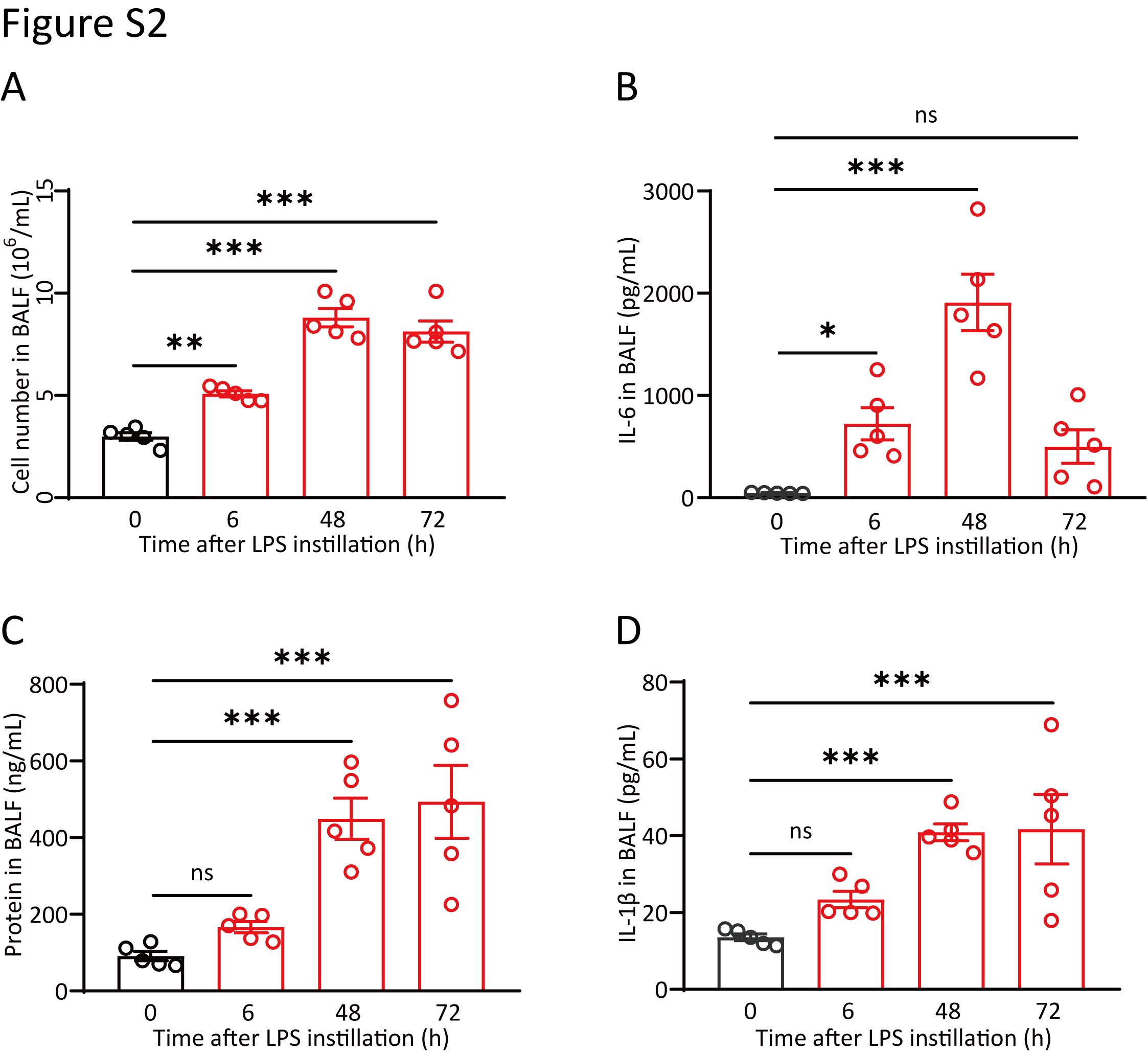

Supplement: Supplementary file 2 [file Image_2.tif]

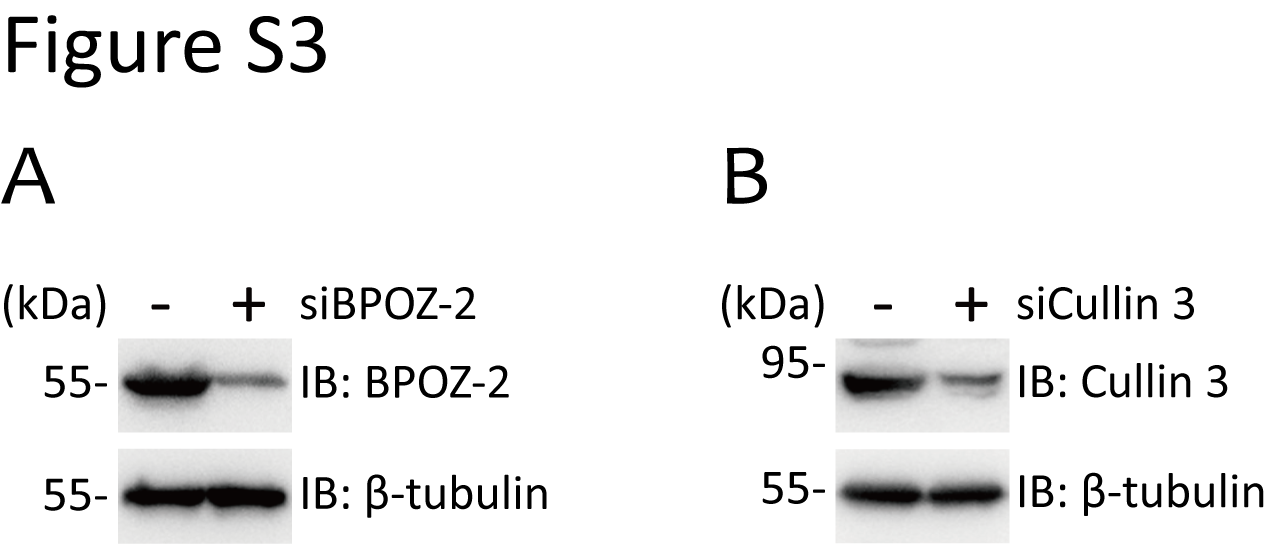

Supplement: Supplementary file 3 [file Image_3.tif]
