# Supplementary material for: BPOZ-2 is a negative regulator of the NLPR3 inflammasome contributing to SARS-CoV-2-induced hyperinflammation
Source: Front Cell Infect Microbiol. 2023 Mar 2;13:1134511. doi: 10.3389/fcimb.2023.1134511 (PMC10019892; doi:10.3389/fcimb.2023.1134511)
Supplement: Supplementary file 4 [file Table_1.docx]

Table S1 Sequences of primers used in this study

| Primers | Gene name | Sequence (5'to3') |
| --- | --- | --- |
| qhBPOZ-F | *BPOZ-2* (human) | CGAGGCCAACACCTTCGATG |
| qhBPOZ-R |  | TGACCTGCTTGTAATCGCGTA |
| qhActin-F | *Actin* (human) | CGGCACCACCATGTACCCTG |
| qhActin-R |  | ACACGGAGTACTTGCGCTCA |
| qmBPOZ-F | *BPOZ-2* (mouse) | GTGCGCTACCTGTTGGCTAAT |
| qmBPOZ-R |  | GAACGGCTTTCCGTGTACCA |
| qmActin-F | *Actin* (mouse) | CATGTACCCAGGCATTGCTGAC |
| qmActin-R |  | TCCACACAGAGTACTTGCGCTCA |
| qmNLRP3-F | *NLRP3* (mouse) | CTAAGCAGCCTCATCCGAA |
| qmNLRP3-R |  | GCAGTTTCTCCAAGGCTACCG |
